# Bone and body composition analyzed by Dual-energy X-ray Absorptiometry (DXA) in clinical and nutritional evaluation of young patients with Cystic Fibrosis: a cross-sectional study

**DOI:** 10.1186/1471-2431-9-61

**Published:** 2009-09-28

**Authors:** Vincenzina Lucidi, Carla Bizzarri, Federico Alghisi, Sergio Bella, Beatrice Russo, Graziamaria Ubertini, Marco Cappa

**Affiliations:** 1 Unit of Cystic Fibrosis, Department of Pediatric Medicine - Bambino Gesù Children's Hospital, IRCCS, Rome, Italy; 2 Unit of Endocrinology, Department of Pediatric Medicine - Bambino Gesù Children's Hospital, IRCCS, Rome, Italy

## Abstract

**Background:**

the improved general therapy has led to reduced morbidity and mortality from Cystic Fibrosis (CF), and bone status may have a potentially greater clinical impact.

We investigated the correlation between the severity of the clinical condition, bone status and body composition parameters, in a group of children and young adults with CF.

**Methods:**

we measured lumbar spine bone density and total body composition by dual energy x-ray absorptiometry (DXA) in 82 consecutive CF patients (42 males; median age: 13 years - range: 5-30). Eighty-two healthy subjects, matched for age, gender, height and pubertal stage were recruited as a control group.

**Results:**

37 patients (45.1%) had a normal bone mineral density (BMD). A BMD reduction were observed in 45 (54.8%) patients. Lumbar spine Z score was positively related to Body Mass Index (BMI) and a higher Shwachman-Kulczycki score, and negatively related to Crispin-Norman score. A positive and significant correlation was also observed between lumbar spine Z score and total body composition.

**Conclusion:**

a significant BMD reduction can be present early in CF children and adolescents. A careful follow up of bone status is required starting in childhood.

## Background

With the dramatic improvement in life expectancy for cystic fibrosis (CF) patients over the past 25 years, attention has turned towards the complications of this disease. In particular, research has been directed towards factors which may maximize both survival and quality of life in the longer term [1,2]. Osteopenia, which is commonly found in adult CF patients exacts a considerable toll through fragility fractures; back pain and kyphosis can cause a further deterioration in respiratory status [3-5]. Many reasons have been suggested for the observed reduction in bone mineral density (BMD): small bone size, poor nutritional intake, pancreatic insufficiency, calcium and vitamin D malabsorption, reduced levels of physical activity, corticosteroid usage, pubertal delay, chronic respiratory infection and elevated levels of osteoclast activating cytokines [6]. Failure to accrete a bone mass at an appropriate rate, rather than bone loss, has been hypothesized to play the major role [7].

Until the late 1990s, over 30% of CF subjects were reported to have a very low BMD, with lower values in men than in women [8]. The reduction in BMD seems to evolve during adolescence, and becomes more evident in adulthood. More recently, some studies [9,10] have reported overall normal BMD in well-nourished CF subjects. A few authors have described that ΔF508 mutation in the homo- or heterozygous state could represent an independent risk factor for reduced bone density in CF [11].

The purpose of this study was to analyze BMD and body composition by DXA in a large heterogeneous group of children and young adults with CF.

We investigated the correlation between various indicators of clinical condition (especially nutrition and lung function) and bone status.

## Methods

### Subjects

The study was a cross-sectional, observational evaluation of children and young adults with CF. All the patients, aged between 5 and 30 years, attending the Cystic Fibrosis Unit at the Bambino Gesù Children's Hospital of Rome, were invited to participate in the study, without obligation and without specific selection criteria. The study period extended from July 2007 to July 2008. Eighty-two (42 males, 40 females) consecutive patients underwent the evaluation described below. Median age was 13 years (range 5-30). Twenty-seven patients (32.9%) were children (aged 5 years to puberty), 45 (54.9%) were adolescents (from the onset of puberty to 18 years), and 10 (12.2%) were young adults (older than 18 years). The diagnosis of CF had been confirmed by the sweat test and by CF gene mutation analysis in all patients. All patients were on unrestricted high caloric diet (120% of the daily caloric intake), in accordance with standard recommendations for CF care. No patient used supplemental nocturnal enteral feeding. All patients took an oral multivitamin preparation, including 400 UI/day of vitamin D; no oral calcium supplement was used. In our Unit, oral glucocorticoids are used only in cases of allergic bronchopulmonary aspergillosis (ABPA), severe asthma, and in seriously ill patients. During the previous 2 years, no patient had been treated with oral steroids for more than 15 days. All patients were receiving inhaled steroids; cumulative dose was calculated in mg as the product of mean daily dose × 365 × duration in years. Medical history for bone fractures was negative in all patients. All subjects underwent plain chest X-rays (lateral projection) to exclude unknown vertebral fractures. No patient was taking bone sparing drugs. Clinical and genetic features of CF subjects are summarized in Table 1.

**Table 1:** Clinical and genetic features of CF group

	**Number (%)**	**males**	**females**
Homozygous for ΔF508 mutation	16 (19.5)	9	7

Heterozygous for ΔF508 mutation	39 (47.6)	22	17

No allele for ΔF508 mutation	27 (32.9)	12	15

Pancreatic insufficiency	80 (97.6)	38	42

CF-Related Diabetes	14 (17.1)	6	8

CF-Related Liver Disease*	11 (13.4)	6	5

*:According to Colombo et al [35]

The areal BMD (g/cm^2^) measured by DXA is particularly misleading when measured in growing patients, as it underestimates the true density value for smaller bones and overestimates it for larger bones [12]. Furthermore, it is well known that patients with CF might present growth retardation and pubertal delay, and consequently skeletal size and mineralization may be decreased. In order to minimize the influence of bone size on DXA measured bone mineral content (BMC) and BMD, we intentionally recruited 82 control subjects (42 males) matched for age, gender, pubertal stage and height. For every patient we selected one control subject, matching criteria were: age ± 12 months, height ± 10 cm, and the same pubertal stage. Control subjects were recruited from amongst the healthy children attending the outpatient clinic of our Hospital. Young adult control subjects were students or members of the staff. The study was approved by the Bambino Gesù Institutional Research Committee and written informed consent was obtained prior to participation from patients and/or parents.

## Methods

Weight was assessed by a digital scale, height by a Harpenden stadiometer. Body mass index (BMI) was calculated from the ratio of weight/height^2 ^(kg/m^2^). Due to the presence of pediatric subjects, BMI was also expressed as BMI-Z score, using the Italian reference data [13]. Pubertal status was determined using breast and pubic hair stages in girls, testicular and pubic hair stages in boys, according to the Tanner criteria [14]. Bone status and body composition were analyzed by DXA (DXA Hologic QDR, Waltham, Mass.) equipped with standard density software. Total body fat mass (excluding head) and lean mass were analyzed by DXA and expressed in grams. BMD (g/cm^2^) was assessed at the lumbar spine (L1-L4); the results were expressed as Z score for age, sex and ethnicity according to the reference data given by the Hologic software. Z scores were calculated by subtracting the sex- and age-specific population mean BMD from the CF subject's BMD; this value was then divided by the SD of the sex- and age-specific mean. The areal BMD measurements do not take into account that the actual bone volume is strictly related to body size, a particularly important aspect when evaluating a growing skeleton. A reduced BMD can be the result of a real reduction in BMD and/or the result of a small bone size [12]. To reduce these discrepancies, we also calculated lumbar spine bone mineral apparent density (BMAD), by dividing the vertebral BMC (expressed in grams) by the estimated volume, assuming that vertebral bodies are cubical [15]. The BMAD represents the estimated cortical and trabecular volumetric (g/cm^3^) bone density within the estimated envelope of the vertebral body.

The definition criteria of osteoporosis in pediatric age is not completely established, due to the lack of the linear correlation, seen in adults, between the severity of bone demineralization and the risk of fractures. Even if our study population also comprises a few young adult subjects, we arbitrarily used the standards recently proposed for pediatric subjects to define bone density reduction [12]. We considered **normal BMD **a lumbar spine Z score >-1, **mild BMD reduction **a lumbar spine Z score lower than -1.0 but higher than -2, **severe BMD reduction **a lumbar spine Z score lower than -2.0. Pulmonary function was assessed by forced expiratory volume in one second and expressed as percentage of expected for height (FEV%). Disease severity was globally evaluated by the Shwachman-Kulczycki score, in which four separate aspects are assessed: activity, physical examination, nutrition and chest roentgenogram. Each category was given an equal value of 25 points, each item being rated on a 5-point scale, giving a total score of 100 points, where a higher score indicated better health [16]. Crispin-Norman radiological scoring system was used to evaluate the degree of lung damage. It describes radiological lung lesions: air trapping, linear markings, nodular-cystic lesions, large lesions, general severity, attributing from 1 to 4 or 5 points to describe the increasing severity. Higher scores are associated with increased severity with a total of 25 demerit points [17].

### Statistical analysis

The statistical analyses were performed using the SPSS software, version 6.1 for Windows (SPSS Inc., Chicago, Illinois, USA). The symmetrically distributed data are expressed as mean ± 1SD. Those variables that showed evidence of being asymmetric are presented as median. Linear regression analysis was used to study the relation between lumbar spine Z score and body composition parameters, and to analyze the correlation between bone status and the indexes of disease severity. T-test and One-way Anova were used to calculate differences between means. The Mann-Whitney test was used to compare notably asymmetric variables. P < 0.05 was considered statistically significant.

## Results

**Figure 1:**
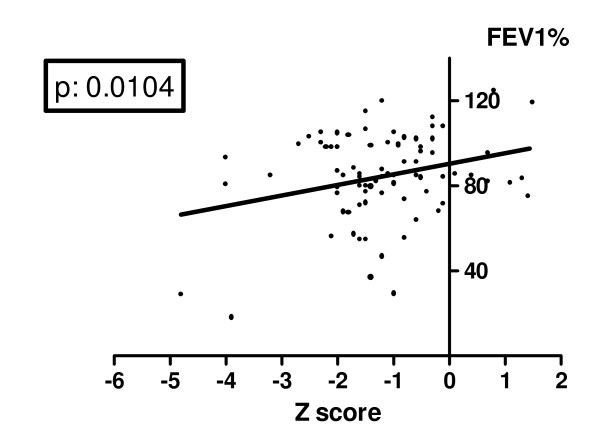
**Correlation between lumbar spine Z score and FEV1%**.

**Figure 2:**
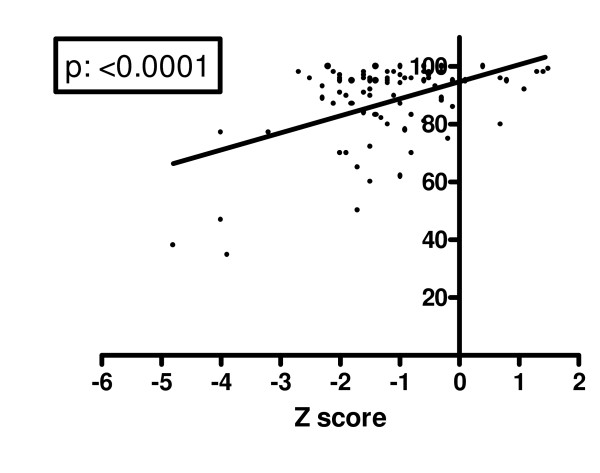
**Correlation between lumbar spine Z score and Shwachman score**.

**Figure 3:**
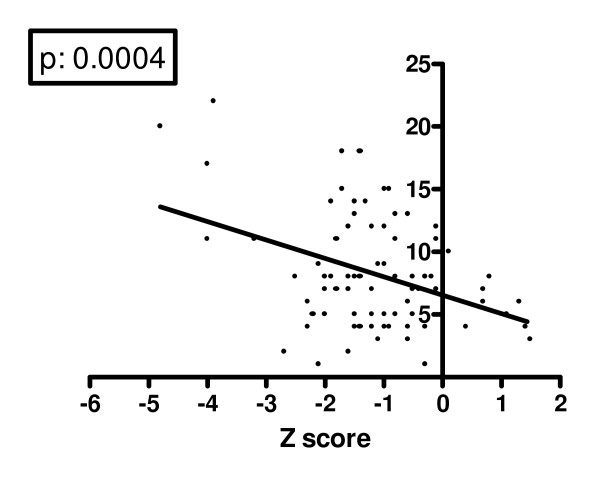
**Correlation between lumbar spine Z score and Crispin-Norman score**.

Mean FEV1% predicted in our population was 84.7 ± 20.5%, four patients (4.8%) had a severe lung disease with a FEV1% < 40%. Mean Shwachman-Kulczycki score was 87.64 ± 14.07, mean Crispin-Norman score was 8.32 ± 4.5. Mean cumulative dosage of inhaled steroids was 721 mg ± 185. Mean lumbar spine BMD Z score and mean lumbar spine BMAD were significantly different in CF subjects and control group; auxological characteristics and bone status of the two groups are shown in Table 2. Thirty-seven subjects had a normal BMD, with a Z score higher than -1 (45.1% - 20 males and 17 females). A mild BMD reduction (with lumbar spine Z score lower than -1.0, but higher than -2.0) was documented in 33 subjects (40.2% - 17 males, 16 females). A condition of severe BMD reduction, with a lumbar spine Z score less than -2, was evident in 12 patients (14.6% - 6 males, 6 females). Median age was not statistically different in the two groups: 13 years (range: 5-30) in normal bone density group, 12 years (range: 5-28) in the group with BMD reduction (Mann-Whitney test). Table 3 shows the different distribution of BMD Z score in prepubertal and pubertal subjects. No subject had a history of fractures and unknown vertebral fractures were excluded by plain chest X-rays in all of them. The cumulative dosage of inhaled steroids in the patient group with severe BMD reduction (BMD <-2) was higher than in the other patients, but the difference did not reach the statistical significance (p = 0.06). There were no statistically significant differences in bone density among patients homozygous for ΔF508 mutation, heterozygous for the same mutation, or carrying different mutations (p: 0.8844). Lumbar spine BMD Z score was negatively and significantly related to FEV1% (p: 0.0104 - figure 1). The severity of BMD reduction seemed to be directly correlated to the clinical severity of the chronic illness (figures 2 &3): lumbar spine BMD Z score was positively related to higher Shwachman-Kulczycki score (p: < 0.0001); to further confirm these data lumbar spine BMD Z score was also negatively related to Crispin-Norman score (p: 0.0004).

**Table 2:** Auxological evaluation and bone status in CF, compared to the control group

	**CF patients**	**Control subjects**	**p**
N°	82	82	

Age (mean ± SD)	13.5 ± 5.6	12.9 ± 5.9	0.59

Height Z score	-0.42 ± 0.96	-0.46 ± 0.95	0.09

BMI Z score	-0.004 ± 1.28	0.11 ± 1.01	0.1

Lumbar spine BMD (Z score ± SD)	-1.18 ± 1.16	-0.67 ± 0.92	< 0.0001

Lumbar spine BMAD (mean ± SD)	0.17 ± 0.03	0.19 ± 0.02	0.002

**Table 3:** Different distribution of BMD Z score in prepubertal and pubertal subjects

	**Mild BMD reduction**	**Severe BMD reduction**	**Normal BMD**
Prepubertal subjects (%)	14 (50%)	3 (10.7%)	11 (39.3%)

Pubertal subjects (%)	19 (35.2%)	9 (16.7%)	26 (48.1%)

**Figure 4:**
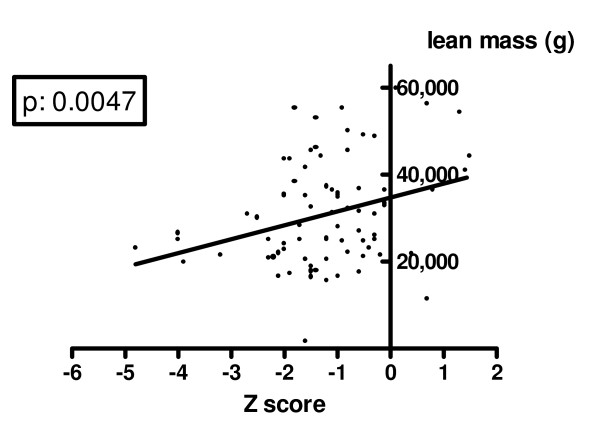
**Correlation between lumbar spine Z score and lean mass**.

**Figure 5:**
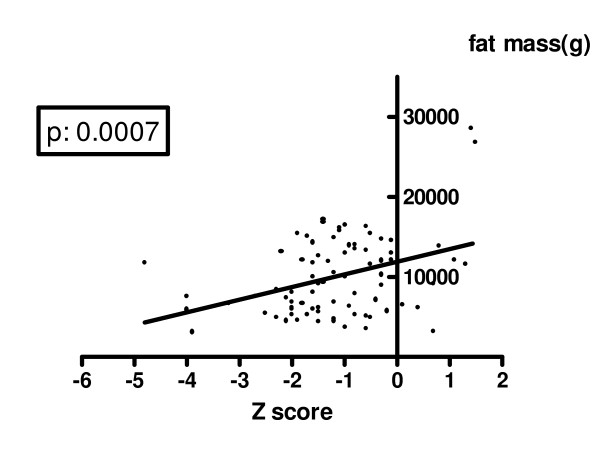
**Correlation between lumbar spine Z score and fat mass**.

Analyzing the indicators of nutrition and the body composition parameters, we found that BMD Z score was significantly reduced in the group of subjects (n = 12) with BMI SDS <-1 in comparison with the group of subjects with BMI SDS >-1 (-2.00 ± 1.59 vs -1.04 ± 1.02, p = 0.0076). A positive significant correlation was evident between lumbar spine BMD Z score and BMI (p: 0.0001). A similar but not significant positive correlation was found with BMI-Z score (p: 0.09). The analysis of body composition parameters showed that lumbar spine BMD Z score was positively and significantly related both to total lean mass (p: 0.0047 - figure 4) and to total fat mass (p: 0.007 - figure 5) expressed in grams. Table 4 shows clinical characteristics and bone status of the 12 patients with severe BMD reduction.

**Table 4:** Clinical characteristics and bone status of the 12 patients with severe BMD reduction.

**Patient**	**gender**	**age**	**Height** **Z score**	**BMI** **Z score**	**FEV1%**	**Lumbar spine** **BMD** **Z score**	**Lumbar spine BMAD (g/cm^3^)**	**Additional causes** **of BMD reduction**
1	female	18	-2.95	-2.30	29	-4.8	0.14	Severe bilial cirrhosis, CFRD

2	male	13	-2.42	-1.22	80.5	-4	0.12	Patient living in a developing country, liver disease, CFRD

3	female	15	-1.75	-2.01	93.4	-4	0.12	Late CF diagnosis (14 yrs), celiac disease, CFRD

4	male	10	-1.22	1.35	18	-3.9	0.11	Right lung exeresis at age 4

5	female	15	-2.7	0,25	84,8	-3.2	0.13	Short bowel syndrome

6	male	13	-1.1	0,11	99,3	-2,7	0,12	Poor compliance

7	male	13	0.03	-0.48	102,9	-2.5	0.12	Poor compliance

8	female	11	-1.52	1.26	100	-2.3	0.12	Cow milk allergy

9	male	11	-0.24	-0.80	105.2	-2.3	0,12	Low social condition

10	female	9	+0.2	0.84	98	-2.2	0.11	No identified cause

11	female	7	-1.11	-0.26	98.1	-2.1	0. 11	No identified cause

12	male	11	-0.98	-0.89	56.3	-2.1	0.12	Prolonged steroid therapy for ABPA, CFRD

CFRD = Cystic Fibrosis Related Diabetes; ABPA = Allergic BronchoPulmonary Aspergillosis

## Discussion

Several studies have described low bone density in CF adults, which might represent a potential consistent risk of fractures [18-21]. Data in pediatric age have been conflicting and, until recently, unconvincing. Bhudhikanok et al [22] reported a significant reduction in total body and areal BMD for both male and female CF patients. Gronowitz [23] reported a normal annual increase of bone mineral density during two years in patients with CF. More recently, Conway et al [24] showed a low BMD in 17% of their study population, which was reduced to 9% after correction for body size by BMAD calculation. None of these studies used an adequate control group of healthy subjects. However, the Conway group results are similar to those reported by Kelly et al [25] in a controlled study evaluating 82 patients (aged 8-18 years) with mild-moderate CF and pancreatic insufficiency. The Kelly study showed that BMD deficits in children are largely dependent on the effect of growth and body composition on bone health, as BMD values normalized after adjustment for height and lean body mass standard deviation scores. Conversely, marked differences have been observed by Bianchi et al in the Milan pediatric CF centre [26]. Longitudinal evaluation over 12-24 months of a sample of 136 young patients (aged 3-24 years) showed that a lumbar spine Z score less than -1 was present in 66%, independently of sex, age and correction for body size (BMAD). Compared to control group, CF patients manifested also a reduction of fat mass and muscle mass (expressed as fat free mass). The severity of bone mass reduction was related to pulmonary function, steroid dose, and the presence of advanced liver disease with portal hypertension. Similarly, Buntain et al [27] analyzed bone mass accrual over 2 years in 85 CF subjects, aged 5-18 years, and showed that BMD gains over time were significantly less than in controls and positively associated with total lean tissue mass. Lung function parameters were significantly associated with BMD gains during adolescence.

Our study confirms the correlation between BMD, clinical scores, lung function and body composition seen in literature [24,26,27]; our results are similar to those of Bianchi et al [26], but we found a lower percentage of patients with BMD reduction (54.8% versus 66%). In particular in our study group, the percentage of patients with severely reduced BMD was significantly lower (14.6% versus 34%). The difference is probably due to the lower percentage of patients with severe lung disease in our study group (4.8% versus 11.7%) and to the different control group used, as the healthy subjects were matched for height only in our study. In contrast with our results, normal BMD values have been reported in CF patients compared with healthy height matched subjects [10,28]. Overall, these discrepancies among studies are probably due to differences in sample size and composition of the study population, and influenced by the severity of lung disease and/or the presence of CF-related complications, the use of steroids, comparison with a control group and data correction for body size.

In our study group anthropometric measures were not dramatically reduced (mean BMI Z-score: -0.004 ± 1.28; mean height Z score: -0.42 ± 0.96). The mean FEV1 in our population was 84.7 ± 20.5% predicted, indicating that our CF patients had on the whole a relatively mild disease. Lumbar spine BMD Z score showed a significant positive correlation with fat and lean mass, confirming that bone status is strictly related to nutrition. Furthermore, BMD Z score was significantly reduced in the group of subjects (n = 12) with BMI SDS <-1 in comparison with the group of subjects with BMI SDS >-1 (-2.00 ± 1.59 vs -1.04 ± 1.02, p = 0.0076). Due to the small size of the group of patients we could not analyze the independent effect of reduced weight and low lean mass on BMD. In our study, lumbar spine BMD Z score is significantly reduced in CF patients, also in prepubertal subjects (see table 3). This finding is strongly supported by BMAD calculation and the comparison of our population with a height matched control group. These results contrast with a few studies which suggested that BMD is normal in prepubertal subjects [27] and that bones are smaller but normally mineralized, mostly in well nourished children [28-30]. However, we have to take into consideration the small size of our control group, caused by the difficulty in identifying appropriated control subjects, especially for CF patients with significant short stature an pubertal delay.

The mean age of our patients with normal BMD is not statistically different from the age of patients with BMD reduction, indicating that bone mass reduction can be present early, during childhood and adolescence, the bone status being conditioned by nutrition and lung function in all ages. The cross-sectional structure of our study did not permit to evaluate, over the years, whether disease severity progressively affects bone mineral status, as observed by others [8].

In our study group no subject had a history of fractures or a detection of unknown vertebral fractures at plain chest X-rays. These data confirm that the linear correlation, seen in adults, between the severity of bone demineralization and the risk of fractures is not evident in pediatric age. Consequently, it should be possible to improve poor bone status in CF children and adolescents with an appropriate treatment of the disease. In our study no patient had been treated with oral steroids for more than 15 days during the previous 2 years; however, all patients used inhaled corticosteroids. Some authors reported a negative association between inhaled corticosteroids and bone mass accrual in children and adolescents with asthma [31] but such an association is still uncertain in CF patients [24-26]. We found no significant difference in cumulative dosage of inhaled steroids comparing patients with severe BMD reduction (BMD <-2) with those with BMD >-2; however, we cannot exclude an influence of continued inhaled steroids on bone mass reduction of our CF patients.

The work of King et al [11] raised the possibility that mutations in CFTR gene may be responsible, at least in part, for low bone density in CF cohorts. The study reported for the first time a direct link between osteoporosis in CF patients and the presence of ΔF508 mutation. No association was found in our population, in accordance with other authors [24,32]; however, our data are not conclusive due to the small size of our series.

We did not study the vitamin D serum levels in our population; vitamin D deficiency is common in CF patients but its role in bone disease is still debatable [24,33,34].

## Conclusion

Our results confirm that the degree of CF severity is correlated with an increased risk of low bone mineral density. A bone mass reduction can be evident early in CF. CF patients with mild and severe BMD reduction had worse pulmonary function, body composition and nutritional status. In CF patients, nutritional evaluation should be complemented by the analysis of bone status and body composition by DXA. At this point in our understanding of CF, maximizing patients' nutritional status, encouraging and maintaining an active lifestyle, and minimizing the progression of chronic suppurative lung disease are essential steps to optimize an individual's potential to attain maximal peak bone mass and thereby reduce the risk of future fractures.

## Competing interests

The authors declare that they have no competing interests.

## Authors' contributions

VL and MC had primary responsibility for protocol development, patient screening, enrollment, and contributed to the writing of the manuscript. CB contributed to patient screening and assessment, and to the writing of the manuscript. FA contributed to the writing of the manuscript. SB, BR and GMU contributed to patient screening and assessment. All authors read and approved the final manuscript.

## Pre-publication history

The pre-publication history for this paper can be accessed here:


